# Majority Group Members' Negative Reactions to Future Demographic Shifts Depend on the Perceived Legitimacy of Their Status: Findings from the United States and Portugal

**DOI:** 10.3389/fpsyg.2018.00079

**Published:** 2018-02-13

**Authors:** H. Robert Outten, Timothy Lee, Rui Costa-Lopes, Michael T. Schmitt, Jorge Vala

**Affiliations:** ^1^Department of Psychology, Trinity College, Hartford, CT, United States; ^2^Institute of Social Sciences, University of Lisbon, Lisbon, Portugal; ^3^Department of Psychology, Simon Fraser University, Burnaby, BC, Canada

**Keywords:** demographic shifts, perceived legitimacy of status, majority, minority, United States, Portugal

## Abstract

Using concepts from social identity theory (Tajfel and Turner, 1979), we examined whether racial/ethnic majority group members' reactions to future demographic shifts is a function of the degree to which they perceive their ingroup's higher-status in society to be legitimate. In two studies, participants who varied in the degree to which they perceived their group's status to be legitimate were either exposed to real projections for 2060 (i.e., large decline in proportion of population that is the “majority” group), or fake projections for 2060—that resembled current figures (i.e., small decline). In Study 1, White Americans who perceived their status to be highly legitimate expressed greater intergroup threat, and negative feelings (anger and fear) toward minorities after exposure to projections with a large decline in the relative size of the White American population. In contrast, demographic shift condition had no effect on intergroup threat and negative feelings toward minorities among White Americans who perceived their status to be relatively illegitimate; negative feelings and threat remained low across both conditions. Similarly, in Study 2, ethnic Portuguese people in Portugal exposed to projections in which there was a large decline in the relative size of the ethnic Portuguese population experienced more intergroup threat and expressed a greater desire to engage in anti-immigration behaviors. The effect of demographic shift condition on intergroup threat and anti-immigration behaviors was stronger among ethnic Portuguese who perceived their status to be legitimate compared to ethnic Portuguese people who perceived their status to be relatively illegitimate. These results highlight that across different cultural contexts, majority group members' beliefs about the legitimacy of intergroup relations can affect their reactions to the prospect of increased diversity.

## Introduction

Many Western countries are projected to be considerably more racially/ethnically diverse by the middle of the twenty-first century (Browne, 2000). In the United States and Canada racial minorities already comprise a larger share of the population than Whites in dozens of major cities (e.g., Vancouver and New York). These cities have been dubbed majority-minority areas—or places where the racial/ethnic majority comprise less than half the population (Frey, 2011; Jedwab, 2016). Western Europe is also becoming more diverse, albeit more slowly (Browne, 2000). London, England is one of the few major European cities that has been designated a majority–minority area. White people of British descent now comprise <50% of the city's population (BBC, 2012). Over the last decade or so, the looming declines in the relative size of racial/ethnic majority groups in western nations have started to garner media attention. Some social and political commentators in the media have speculated about what future demographic shifts might mean for intergroup relations. Some commentators have suggested that there will be increased resentment among majority group members (see Browne, 2000); others argue that majority group members have been “surprisingly chill” with demographic changes thus far and that this bodes well for managing increased diversity in the future (Wilkinson, 2015). Only recently have social scientists started examining how peoples' knowledge of these future changes can affect current intergroup relations. Specifically, social psychologists have begun to experimentally test how being made aware of these impending racial/ethnic demographic shifts affect majority group members' current feelings and attitudes (e.g., Outten et al., 2012; Major et al., 2016).

Recent studies conducted in North America have found that reminding White people of impending demographic shifts can cause them to experience heightened threat and prejudiced feelings toward minorities (e.g., Outten et al., 2012; Craig and Richeson, 2014). For example, Outten et al. (2012, Study 2) exposed White Canadians to either real demographic projections for the city of Vancouver in 2058, where Whites are expected to make up roughly 27% of the population, or fake future projections—which were similar to demographic figures from 2008, in that Whites still made up slightly more than 50% of the population. Afterwards participants completed measures of anger and fear toward minorities and intergroup threat—or appraisals of the degree to which diversity posed a threat to White Canadians. The researchers found that compared to White Canadians exposed to projections in which they were still a numerical majority, participants who viewed projections where White Canadians were a numerical minority expressed more anger and fear toward racial minorities, and greater intergroup threat. In the U.S., reminding White Americans about future declines in their relative group size is associated with greater anti-immigrant attitudes (Major et al., 2016) and greater explicit and implicit prejudice toward racial minorities (Craig and Richeson, 2014). These negative psychological reactions suggest that majority group members use relative group size to make inferences about their group's standing in society. Thus, perceived future decreases in relative group size can constitute an identity threat to majority group members, as they can signal a potential loss of relative dominance or status over racial/ethnic minorities. In turn, the perceived instability of status conveyed by demographic shifts can lead majority group members to feel threatened and express prejudice toward minorities (Outten et al., 2012; Craig and Richeson, 2014).

In sum, recent studies conducted in North America suggest that reminders of demographic shifts can affect majority group members' attitudes and emotional responses. What remains unclear is the psychological and cultural boundary conditions of these negative reactions to future demographic shifts. It is unlikely that all majority group members respond negatively to these anticipated changes. Particular psychological factors might increase or decrease the likelihood of people responding negatively. For example, social identity theory (Tajfel and Turner, 1979) postulates that members of dominant groups who perceive their ingroup's current higher-status position in society as legitimate, should be more prone to react negatively toward lower-status outgroups in response to societal conditions that signal the loss of dominance. As such, one might expect majority group members who perceive their status as legitimate to be more threatened by diversity and to respond more negatively toward minorities, compared to majority group members who perceive their status to be illegitimate. Furthermore, studies on majority group members' responses to anticipated demographic changes have been limited to Canada and the U.S., even though other western nations are expected to become more diverse in the coming decades. Thus, it is unclear whether the findings obtained in North America generalize to other cultural contexts. In an attempt to address these issues, we conducted a study in the U.S. and another in Portugal, to examine if perceived legitimacy of status moderates majority group members' negative psychological responses to future demographic shifts.

## Future demographic changes and the role of perceived legitimacy of status

According to SIT (Tajfel and Turner 1979), belonging to a high-status group contributes to a positive social identity and as such people might be motivated to protect their dominant position. The security of an existing status hierarchy—in terms of its *stability* and *legitimacy*—are key determinants of whether members of high-status groups feel threatened, and in turn protect their group by reacting negatively to lower-status outgroups. Stability refers to the degree to which an existing social hierarchy appears to be changeable or unchangeable. For members of high-status groups, intergroup events like shifting racial/ethnic demographics that signal that their relative social standing is unstable, can be threatening and foster negative feelings toward lower-status outgroups (e.g., Outten et al., 2012; also see Caricati and Sollami, 2017).

Legitimacy refers to the extent that one's higher-status is fair or unfair. According to SIT (Tajfel and Turner, 1979), when status differences are perceived as unfair or illegitimate, members of high-status groups are less likely to feel threatened by lower-status outgroups or behave negatively toward them. Conversely, when members of high-status groups perceive their status as legitimate they become more sensitive to threats to it and are more likely to react negatively to lower-status groups (e.g., LeBlanc et al., 2015). Thus, negative reactions to an unstable future can be exacerbated by perceptions that their ingroup's current status is legitimate. In other words, perceptions of instability and legitimacy can interact such that perceiving that one's legitimate higher-status is unstable can be particularly threatening. Indeed, studies have found that when members of higher-status groups perceive their dominant position as unstable and legitimate, they experience heightened threat about their group's standing and react to lower-status groups with more intense prejudice (e.g., Turner and Brown, 1978; also see LeBlanc et al., 2015). For example, Verkuyten and Reijerse (2008) found a significant interaction between perceived legitimacy and instability on feelings toward minority outgroups in the Netherlands among ethnic Dutch participants. Specifically, the more that ethnic Dutch people saw ethnic relations in the Netherlands as both legitimate and unstable, the more negatively they felt about ethnic minorities. Thus, if we apply SIT (Tajfel and Turner, 1979) to the phenomenon of future demographic shifts (i.e., greater instability), it is reasonable to postulate that majority group members who perceive that their ingroup's status is highly legitimate will respond very negatively to anticipated demographic changes. Conversely, those who can recognize that their ingroup's status is illegitimate to some degree are likely to be less perturbed by future demographic shifts.

Our predictions also resonate with other theoretical perspectives, such as social dominance theory (SDT; Sidanius and Pratto, 1999) and system-justification theory (SJT; Jost et al., 2004), as a major focus of both perspectives is how legitimizing beliefs and ideologies shape responses to group-based inequality. Like social identity theory research, research on SDT and SJT have accumulated a wealth of evidence that legitimacy perceptions moderate responses to intergroup contexts. Where the perspectives differ is in their motivational assumptions. SDT and SJT acknowledge the existence of ingroup-favoring motivations posited by SIT, but they tend to focus on other motivations. SDT assumes a general human motivation (that nonetheless varies between individuals and contexts) for preferring hierarchical systems, that drives acceptance of hierarchy-enhancing legitimizing myths and behaviors that maintain hierarchy (Sidanius and Pratto, 1999). In the case of SJT, it is a general motivation toward justifying social systems that leads to the acceptance of beliefs that legitimize inequality, including hostile responses toward minority groups (Jost et al., 2004). When considering the present context, in which we are hypothesizing about responses from a dominant group, the focal motivations of these three perspectives tend to be aligned, as the interests of dominant groups overlap to a large degree with hierarchy maintenance and system justification. Thus, while we based our predictions on SIT in particular, our predictions are also derivable from SDT and SJT.

## The U.S. vs. Portugal

Currently, our knowledge of how anticipated demographic shifts affect majority group members come from two diverse nations: Canada and the U.S. For example, the U.S. is on the verge of reaching a “tipping point”—or point where the racial/ethnic majority will become a numerical minority by the middle of the twenty-first century. White Americans now constitute roughly 62% of the U.S. population; a figure expected to decline to 44% by the year 2060 (Colby and Ortman, 2015). Thus, prior findings could be interpreted as something unique to North America. To better understand the cultural boundary conditions of how demographic shifts affect majority group members, we conducted research with ethnic Portuguese people in Portugal as well as with White Americans in the U.S.

We selected Portugal as the comparison country because when testing the universality of a psychological process—in our case whether majority group members' negative reactions to future demographic shifts are dependent on the perceived legitimacy of their ingroup's status—it is important to select cultures that differ on some cultural dimensions (see Heine, 2016 for recommendations for conducting cross-cultural research). Indeed, previous cross-cultural studies have found that Americans and Portuguese people differ markedly in their endorsement of several cultural values (e.g., Basabe and Ros, 2005; Yeganeh and May, 2011). For instance, Americans typically hold stronger individualistic values than Portuguese people. That is, Americans tend to prioritize the individual self, personal independence, and achievements more so than Portuguese people. The Portuguese tend to be more collectivistic, in that they are more likely to give precedence to fulfilling obligations to close others and maintaining social harmony. Additionally, Portuguese people generally score higher on values consistent with a commitment to egalitarianism like social justice and equality, compared to people from other nations, including the U.S. (Basabe and Ros, 2005; Schwartz, 2006; Yeganeh and May, 2011).

A cultural difference that is more directly relevant to the current investigation, however, is the fact that Portugal and the U.S. have very different immigration histories. Unlike the U.S., mass immigration to Portugal is a relatively new phenomenon. It was not until after the fall of the dictatorial regime and the independence of the former African colonies in the mid-1970's that Portugal began to experience significant immigration flows. Much of the early immigration flows consisted of Portuguese returnees and African laborers from former colonies like Cape Verde and Angola. In 1980, ethnic Portuguese people accounted for 99.5% of the total population of the country. Today, roughly 90% are ethnic Portuguese (see Peixoto and Sabino, 2009 for a review Portugal's immigration history). However, by 2060 ethnic Portuguese people are projected to make-up 70% of the population (European Commission, 2011). Thus, while Portugal is not expected to reach a “tipping point” within the next 40–50 years, Portugal has become much more diverse in a relatively short period of time, which could represent an identity threat for ethnic Portuguese people, and foster negative feelings toward diversity and hostility toward minorities.

## Overview of studies

In two experiments we examined how majority group members in the U.S. (Study 1) and Portugal (Study 2) responded to real future demographic shifts in which their ingroup's relative size is expected to decline markedly. Consistent with SIT (Tajfel and Turner, 1979), we predicted that thinking of instability in the form of large declines in the relative size of the majority population would lead to greater intergroup threat (Study 1 and 2), anger and fear toward minorities (Study 1) and a willingness to engage in anti-immigration behaviors (Study 2). Furthermore, we expected that negative reactions to future demographic shifts would be moderated by perceptions of legitimacy. Specifically, majority group members who perceive their group's status as legitimate would experience greater intergroup threat and prejudiced responses toward minorities after exposure to projections showing a large decrease in the relative size of their majority group, compared with those who perceive their ingroup's status as relatively illegitimate.

In Study 1, White Americans were either presented with real projections for 2060, in which they become a numerical minority (43.6% of the population), or fake projections for 2060 that resembled figures from 2010 (Whites = 59% of the population). In Study 2, ethnic Portuguese people were either presented with real projections for 2060 in which (ethnic Portuguese = 70% of the population), or fake projections that resembled figures from 2010 (ethnic Portuguese = 90% of the population). In both studies, we first measured the degree to which majority group members perceived their status to be legitimate. Then participants were exposed to one of two projections for 2060 representing either a large decline condition (i.e., real projections) or a small decline condition (i.e., fake projections). Next, participants completed indicators of threat and negative reactions toward minorities. For the experimental manipulations used both studies see Supplementary Materials.

## Study 1

In Study 1, we randomly assigned White American participants to either a condition in which they were shown projections illustrating a large decline in the relative size of the White American population or projections showing a small decline in the relative size of the White American population. We predicted that White Americans exposed to real projections, in which their relative share of U.S. population is expected to decline markedly, would exhibit greater intergroup threat, and greater anger and fear toward minorities. We expected individual differences in perceived legitimacy to moderate the effect of condition on intergroup threat and negative feelings toward minorities. Specifically, compared to White Americans who perceive their status to be relatively illegitimate, White Americans who perceive their status as legitimate would experience heightened intergroup threat and negative feelings toward minorities after exposure to either set of projections. However, intergroup threat and negative feelings toward minorities would be highest among White Americans who perceive their status to be legitimate and are exposed to projections showing a large decrease in the proportion of the U.S. population that is White.

### Methods

#### Participants and ethics statement

Two-hundred White Americans (51% men, *M*_*age*_ = 38.3 years, *SD* = 10.59) were recruited via Amazon Mechanical Turk (Buhrmester et al., 2011) and paid U.S. $3.50. In accordance with the Declaration of Helsinki all participants provided informed consent electronically before beginning the study. The experiment was approved by the Trinity College Institutional Review Board.

#### Materials and procedure

All survey materials in Study 1 were written in English. Participants completed a questionnaire that was created using SurveyGizmo (2017). They were told that they were participating in a study examining memory and recall. First, participants provided consent and completed background questions about their age, gender, legal status in the U.S., household income, and education level. Embedded within the background questions were two questions that assessed perceived legitimacy of status. Next, participants were randomly assigned to either a large decline condition (i.e., real projections) or a small decline (i.e., fake projections). In both conditions, participants were presented with a newspaper article about racial demographic projections. Both articles contained real racial demographic figures for 2010, where White Americans comprised 63.7% of the population and racial minorities comprised 36.3% (see Humes et al., 2011). However, in the large decline condition a second figure showed that Whites would account for 43.6% of the population in 2060, whereas racial minorities would account for 56.4% (Colby and Ortman, 2015). In the small decline condition, the additional figure showed that Whites would account for 59% of the population and racial minorities would account for 41%. We encouraged participants to read the article closely, because they would later complete a recall test based on the article's contents. In actuality, the recall test served as a manipulation check. Prior to the recall test participants completed measures of intergroup threat and anger and fear toward minorities. After completing the dependent measures and the recall test, participants were fully debriefed.

#### Measures

The measure of perceived legitimacy of status was adapted from Weber et al. (2002), whereas the measures of intergroup threat appraisals, anger and fear toward minorities and the manipulation check were adapted from Outten et al. (2012).

##### Perceived legitimacy of status

Two items were averaged to create a perceived legitimacy index (“It is quite justified that White Americans have higher status in society than other racial groups in the U.S.” and “White Americans deserve to have a better standing in society than other racial groups in the U.S.”; *r* = 0.91). The scales ranged from 1 (*strongly disagree*) to 9 (*strongly agree*). Higher scores indicated greater perceived legitimacy of status.

##### Intergroup threat appraisals

Appraisals of intergroup threat were measured using a 3-item scale. The three items included: “My racial group should be worried about its place in the future of the U.S.,” “My racial group should be threatened by growing diversity” and “My racial group will benefit from increasing diversity in the U.S.” α = 0.90. The third item was reverse-coded. Response scales ranged from 1 (*strongly disagree*) to 7 (*strongly agree*). Higher scores indicated greater perceived threat to one's ingroup from growing diversity.

##### Anger and fear toward minorities

Participants were asked how they felt toward racial minorities after reading the article. Specifically, they were asked about the extent to which they felt anger (angry, annoyed, resentful; α = 0.96) and fear (fearful, scared, frightened; α = 0.96) toward minorities. The intergroup emotions scales were constructed by averaging the scores of three individual items that comprised each emotion. The response scales ranged from 1 (*not at all*) to 11 (*extremely*). Higher scores indicated feeling greater anger and fear toward minorities.

##### Manipulation check

After completing statements assessing threat, anger and fear participants were presented with an open-ended manipulation check in which they were asked to recall the percentages that Whites and racial minorities were expected to make-up in 2060.

### Results

Frequency statistics for age, household income and education level for White American (Study 1) and ethnic Portuguese (Study 2) samples are presented in Table 1. The means and standard deviations for all focal variables in Study 1 and Study 2 are presented in Table 2.

**Table 1:** Age, household income, and education frequency statistics for U.S. and Portuguese samples.

**Study 1: U.S. sample (*****N*** = **200)**		**Study 2: Portugal sample (*****N*** = **92)**	
**Age**		**Age**	
<20	0%	<20	10%
20–29	21%	20–29	89%
30–39	41%	30–39	1%
40–49	20%	40–49	0%
50–59	13%	50–59	0%
60+	6%	60+	0%
**Household income (yearly, US dollars)**		**Household income (monthly, Euros)**	
<$23,999	22%	<€458	35%
$24,000–44,999	26%	€458–833	22%
$45,000–69,999	24%	€834–1,167	14%
$70,000–99,999	18%	€1,168–2,083	17%
$100,000+	11%	€2,083–2,999	10%
		€3,000+	2%
**Highest level of education**		**Highest level of education**	
Attended or completed high school	16%	Attending or completed high school	45%
Attend/ed or completed college or university	70%	Completed an undergraduate degree	45%
Attend/ed or completed graduate program	15%	Completed a postgraduate or Master's degree	10%
		Completed a doctorate	1%

*Different categories for household income and level of education were used in the two studies. As such, they are not directly comparable. Household income in the U.S. is typically measured in annual amounts, whereas household income in Portugal is typically measured in monthly amounts. The household income statistics presented in the table reflect those differences. Due to rounding some category totals do not add up to 100%*.

**Table 2:** Means, standard deviations for perceived legitimacy of status, and dependent variables by demographic condition for Study 1 and Study 2.

	**Demographic shift condition**				
	**Small decline (fake projections)**		**Large decline (real projections)**		
	***M***	***SD***	***M***	***SD***	***P***
**STUDY 1: U.S. SAMPLE**					
Perceived legitimacy of status	4.17	2.43	4.36	2.58	0.61
Intergroup threat appraisal	2.86	1.47	3.37	1.90	0.04
Anger toward minorities	1.92	1.56	2.81	2.83	<0.01
Fear toward minorities	1.86	1.46	2.71	2.63	<0.01
**STUDY 2: PORTUGAL SAMPLE**					
Perceived legitimacy of status	4.80	1.94	4.90	2.37	0.83
Intergroup threat appraisal	2.95	0.86	4.27	1.53	<0.001
Willingness to engage in anti-immigrant behaviors	2.60	1.18	3.37	1.44	<0.01

#### Manipulation check

A one-way ANOVA showed that participants' recall test responses varied significantly by condition. Participants in the large decline condition (*M* = 43.86) reported that Whites would comprise a smaller share of the total population in 2060, relative to those in the small decline condition (*M* = 57.17), *F*_(1, 198)_ = 544.56, *p* < 0.001. Also participants in the large decline condition reported that racial minorities would make up a significantly greater share of the population in 2060 (*M* = 56.38), compared with those in the small decline condition (*M* = 42.76), *F*_(1, 198)_ = 521.78, *p* < 0.001.

#### Effect of condition on dependent measures

Next, we tested whether the manipulation of demographic information had different effects on intergroup threat, anger and fear. Consistent with previous research (Outten et al., 2012) participants in the large decline condition reported experiencing greater intergroup threat, *F*_(1, 198)_ = 4.32, *p* = 0.04; anger toward minorities, *F*_(1, 198)_ = 7.13, *p* < 0.01; and fear toward minorities, *F*_(1, 198)_ = 7.46, *p* < 0.01.

#### Perceived legitimacy of status as a moderator

To examine whether perceived legitimacy of status moderated the effect of demographic shift condition (coded: 1 = large decline/real projections and 0 = small decline/fake projections) on our dependent measures (intergroup threat, anger, and fear), we ran three simple moderation models using Hayes' (2013; Model 1) PROCESS macro for SPSS. This allowed us to test if the effect of condition on intergroup threat, anger and fear varied as a function of perceived legitimacy of status, and provided us with effects at different levels of perceived legitimacy (i.e., conditional effects: ± 1 *SD* from the mean). If the 95% confidence intervals generated do not contain zero, then one can conclude that the conditional moderated effect is significant^1^.

**Figure 1:**
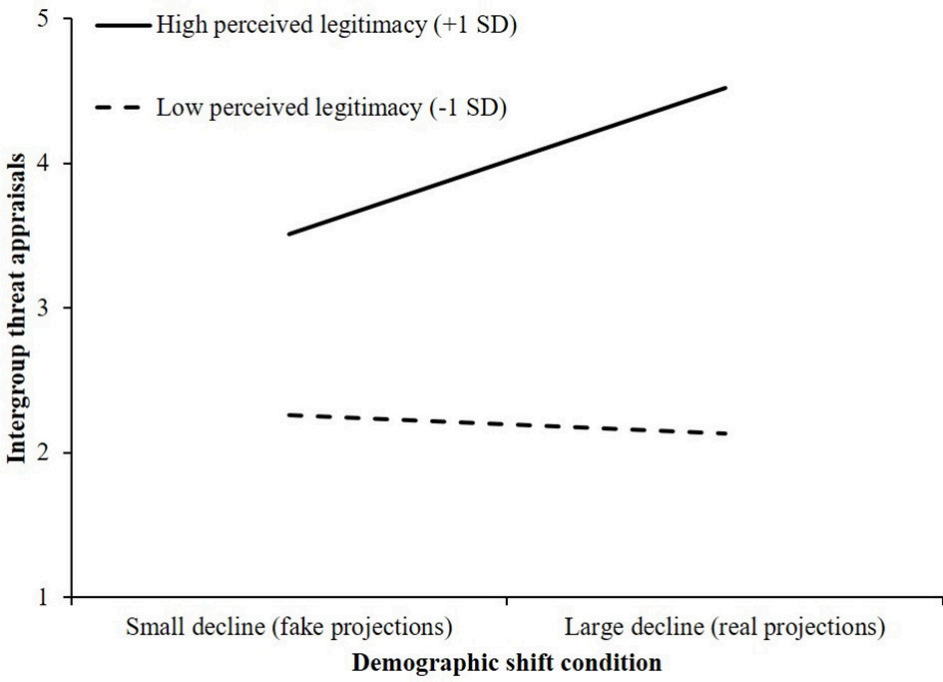
The effect of demographic shift condition and perceived legitimacy of status on intergroup threat appraisals (Study 1).

We found a significant interaction between perceived legitimacy and demographic shift condition on intergroup threat appraisals, *R*^2^ch. = 0.026, *F*_(1, 196)_ = 7.88, *p* < 0.001. Examination of the conditional effects revealed that the effect of condition on intergroup threat was significant for White Americans who perceived their status as legitimate (+1 *SD*; *B* = 1.01, *SE* = 0.29, 95% CI, [0.449, 1.579]). As shown in Figure 1, participants who saw their status as highly legitimate felt significantly more threatened by diversity in the large decline condition, compared with those in the small decline condition. Among White Americans who perceived their status to be relatively illegitimate, condition had no effect on intergroup threat appraisals; threat remained low across both conditions (−1 *SD*; *B* = −0.12, *SE* = 0.28, 95% CI, [−0.683, 0.435]).

**Figure 2:**
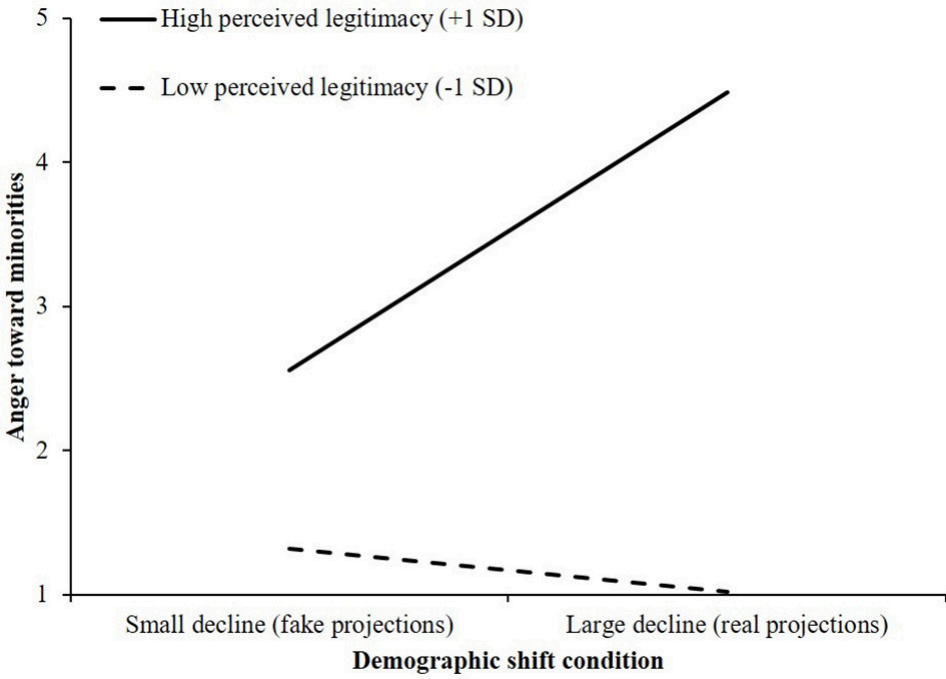
The effect of demographic shift condition and perceived legitimacy of status on anger toward minorities (Study 1).

The proposed interaction between perceived legitimacy and condition on anger toward minorities was statistically significant, *R*^2^ch. = 0.05, *F*_(1, 196)_ = 16.50, *p* < 0.001. Examination of the conditional effects revealed that the effect of exposure to real projections on anger was significant for White Americans who saw their status as legitimate (+1 *SD*; *B* = 1.93, *SE* = 0.39, 95% CI, [1.162, 2.694]). As shown in Figure 2, among White Americans who felt strongly that their status was legitimate expressed significantly more anger toward minorities after viewing projections indicating a large decline in the relative size of White American population, compared with when they viewed projections showing a small decline in the White population. In contrast, for Whites who perceived their status to be relatively illegitimate the amount of anger they felt did not significantly differ across demographic shift conditions (−1 *SD*; *B* = −0.30, *SE* = 0.38, 95% CI, [−1.061, 0.455]). Anger remained low in both conditions.

**Figure 3:**
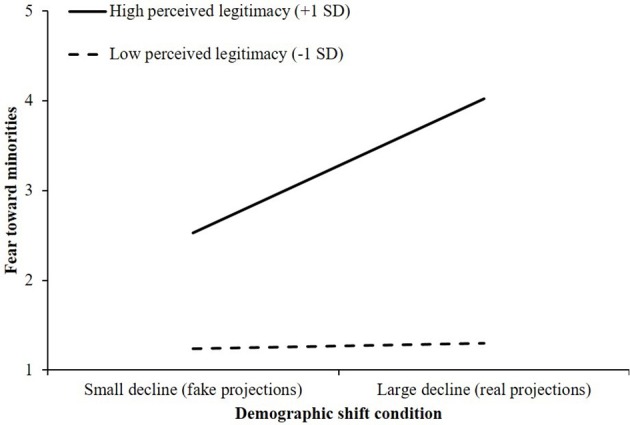
The effect of demographic shift condition and perceived legitimacy of status on fear toward minorities (Study 1).

Finally, we found a significant interaction between perceived legitimacy and the demographic shift condition on fear toward minorities, *R*^2^ch. = 0.03, *F*_(1, 196)_ = 6.98, *p* < 0.01. Inspection of the conditional effects indicated that the effect of exposure to a large decline in the White population on fear was significant. As shown in Figure 3, White Americans who strongly legitimized their current status reported significantly more fear after seeing a large decline in the relative size of the White population, compared to viewing a small decline in the relative size of the White population (+1 *SD*; *B* = 1.50, *SE* = 0.38, 95% CI, [0.742, 2.260]). Conversely, among Whites who appraised their status as relatively illegitimate, condition had no effect on fear toward minorities; fear remained low across both conditions (−1 *SD*; *B* = 0.06, *SE* = 0.38, 95% CI, [−0.689, 0.813]).

### Discussion

Consistent with our predictions, White Americans who viewed projections for 2060 showing a large decline in their racial group's relative size experienced heightened appraisals of intergroup threat, as well as more anger and fear toward racial minorities, compared to Whites who viewed projections showing a small decline. These negative reactions in response to seeing a large decline in the relative size of one's dominant group were greatest among those who perceived their status to be highly legitimate. White Americans who perceived their status as relatively illegitimate did not experience greater intergroup threat and negative feelings toward minorities after viewing projections showing a large decrease in the White population. While these results support SITs (Tajfel and Turner, 1979; also Verkuyten and Reijerse, 2008) contention that among majority group members perceptions of instability and legitimacy are determinants of feeling threatened and reacting negatively toward minority outgroups, it would be prudent to test whether these findings generalize to other cultural contexts. Study 2 attempts to do that by examining similar processes with an ethnic Portuguese sample in Portugal.

## Study 2

In Study 2, we assessed whether exposing ethnic Portuguese people to real population projections showing a large decline in their share of the total population, would lead them to express greater intergroup threat and a stronger desire to engage in behaviors aimed at limiting immigration to Portugal. We also tested if these responses to impending demographic shifts would be moderated by individual differences in perceived legitimacy of status.

It is worth mentioning that Study 2 differed from Study 1 in some respects. First, prior studies examining how majority group members respond to anticipated demographic changes have tended to focus on attitudinal and emotional responses (e.g., Outten et al., 2012; Craig and Richeson, 2014). In an effort to broaden the outcomes examined, we assessed intentions to engage in anti-immigrant behaviors (e.g., attending an anti-immigration protest, voting for anti-immigrant party). We were also interested in assessing behavioral intentions related to immigration because Portugal's population more so than most other nations is currently sustained by immigration. Portugal has one of the lowest fertility rates in the world (Eurostat, 2017). In recent years, the Portuguese government has been more vocal in encouraging immigration, largely due to low fertility rates (Costa and Sousa, 2017). Second, Study 2 utilized a slightly different manipulation of demographic projections. Rather than presenting information broken down by racial groups (i.e., Whites vs. racial minorities), the demographic information presented to participants focused on ethnic heritage (i.e., native Portuguese vs. foreigners and their descendants). This is because unlike the U.S., the Portuguese government does not breakdown population statistics by race, but rather people's countries of origin (e.g., Moreira, 2014; also see European Commission, 2011). As such, it made sense that our projections were consistent with how population projections are usually depicted for Portugal.

Similar to Study 1, ethnic Portuguese participants were randomly assigned to either a condition in which they were shown real projections for 2060 where the proportion of Portugal's population that is ethnic Portuguese declines significantly, or fake projections in which there is a small decline. We predicted that those exposed to projections showing a large decline in the relative size of the ethnic Portuguese population would experience greater intergroup threat and a greater willingness to engage in anti-immigrant behaviors. We expected that ethnic Portuguese people who perceive their status as highly legitimate would experience heightened intergroup threat and a greater willingness to engage in anti-immigrant behaviors after exposure to either set of demographic projections, compared with ethnic Portuguese people who perceive their status to be relatively illegitimate. Appraisals of intergroup threat and anti-immigrant behavioral intentions would be highest among ethnic Portuguese people who were exposed to demographic projections showing a large decline in the relative size of the ethnic Portuguese population and who perceive their status as relatively legitimate.

### Methods

#### Participants and ethics statement

Ninety-seven students from the University of Lisbon were recruited via convenience sampling (38% women, *M*_*age*_ = 22.6 years, *SD* = 6.26). Participants were recruited from undergraduate and graduate classes. They were entered into a draw to win 1 of 2 50€ gift cards from a large national retailer. The final sample consisted of 92 people; 5 participants who did not self-identify as ethnic Portuguese and/or were not from Portugal were excluded from the analysis. Participants' consent was obtained electronically prior to beginning the study in accordance with Declaration of Helsinki. The Institute of Social Sciences at the University of Lisbon—the host institution for this study—did not have an internal review board at the time the study was conceived, but the study was approved by independent scientific experts at the Fundação para a Ciência e a Tecnologia.

#### Materials and procedure

The survey materials that were presented to participants in study 2 were written in the Portuguese. The materials were originally composed in English by the first author. One of the primary investigators and a research assistant, both of whom are bilingual in Portuguese and English, then worked in tandem to ensure accurate translation of the study materials to Portuguese.

The procedure was similar to Study 1. After completing background questions containing a measure of perceived legitimacy of status, participants were randomly assigned to read one of two articles about demographic projections. Across both conditions participants were informed that in 2010 the ethnic Portuguese population was 92.6%, whereas the foreign-born population was 7.4%. However, in the large decline condition (i.e., real projections), participants were also informed that the ethnic Portuguese population would be 70% of the population in 2060, whereas the foreign-born population and their descendants were expected to comprise 30% of the population (European Commission, 2011). For participants assigned to the small decline condition (i.e., fake projections), participants were informed that the ethnic Portuguese population would slightly decrease to 90% in 2060, whereas the foreign-born population and their descendants would increase to 10%. Afterwards participants completed measures of intergroup threat appraisals and anti-immigration behavioral intentions. Participants then took a recall test that served as a manipulation check. Lastly, all participants were fully debriefed.

#### Measures

##### Perceived legitimacy of status

The same perceived legitimacy items from Study 1 were used, but were adapted to the Portuguese context (e.g., “It is quite justified that native Portuguese have higher status in society than foreigners” and “Native Portuguese deserve to have a better standing in society than foreigners”; *r* = 0.94). Scales ranged from 1 (*strongly disagree*) to 9 (*strongly agree*). Higher scores indicated greater perceived legitimacy of status.

##### Appraisals of intergroup threat

The same 3-item measure of intergroup threat appraisals used in Study 1 was adapted to a Portuguese context. The items included, “Portuguese people should be worried about their place in the future of Portugal,” “The Portuguese should be threatened by growing diversity” and “Portuguese people will benefit from increasing diversity in Portugal;” α = 0.78. The third item was reverse-scored. Scales ranged from 1 (*strongly disagree*) to 7 (*strongly agree*). Higher scores indicated greater perceived threat to people of Portuguese descent from growing diversity.

##### Anti-immigration behavioral intentions

Four items were adapted from Tougas et al. (2006) openness to toward immigration measure, to assess willingness to engage in behaviors aimed at limiting immigration to Portugal (i.e., “I would vote for a political party that wanted to limit immigration until unemployment is reduced,” “I would volunteer time to participate in a campaign aimed at getting the government to reduce immigration rates,” “I would sign a petition demanding that the government be more strict in the selection of immigrants,” and “I would attend a demonstration to support stricter immigration laws”; α = 0.85). Items were assessed using a 7-point scales ranging from 1 (*definitely no*) to 7 (*definitely yes*). Higher scores indicated greater anti-immigration behavioral intentions.

##### Manipulation check

Similar to Study 1, participants completed an open-ended manipulation check in which they were asked to recall the projected percentages of ethnic Portuguese people, as well as, the foreign-born population and their descendants in 2060.

### Results

#### Manipulation check

A one-way ANOVA showed that participants' average recall test answers differed significantly by condition. Participants in the large decline condition (*M* = 70.18) reported that ethnic Portuguese would comprise a smaller share of the population in 2060, compared with those in the small decline condition (*M* = 90.49), *F*_(1, 90)_ = 746.68, *p* < 0.001. Furthermore, individuals in the large decline condition reported that foreign-born people and their descendants would make up a larger proportion of the population in 2060 (*M* = 29.33), compared with those in the small decline condition (*M* = 9.47), *F*_(1, 90)_ = 563.29, *p* < 0.001.

#### Effect of condition on dependent measures

Next, we tested whether the manipulation of demographic shifts had different effects on appraisals of intergroup threat and anti-immigration behavioral intentions. Consistent with predictions participants exposed to projections showing a large decline in the relative size of the ethnic Portuguese population experienced a greater appraisal of intergroup threat, *F*_(1, 90)_ = 25.32, *p* < 0.001, and greater endorsement of anti-immigration behavioral intentions, *F*_(1, 90)_ = 7.61, *p* < 0.01.

#### Perceived legitimacy of status as a moderator

Similar to Study 1, we examined whether perceived legitimacy of status moderated the effect condition (coded: 1 = large decline/real projections and 0 = small decline/fake projections) on intergroup threat appraisals and willingness to engage in anti-immigration behaviors by using Hayes' (2013; Model 1) PROCESS macro for SPSS.

**Figure 4:**
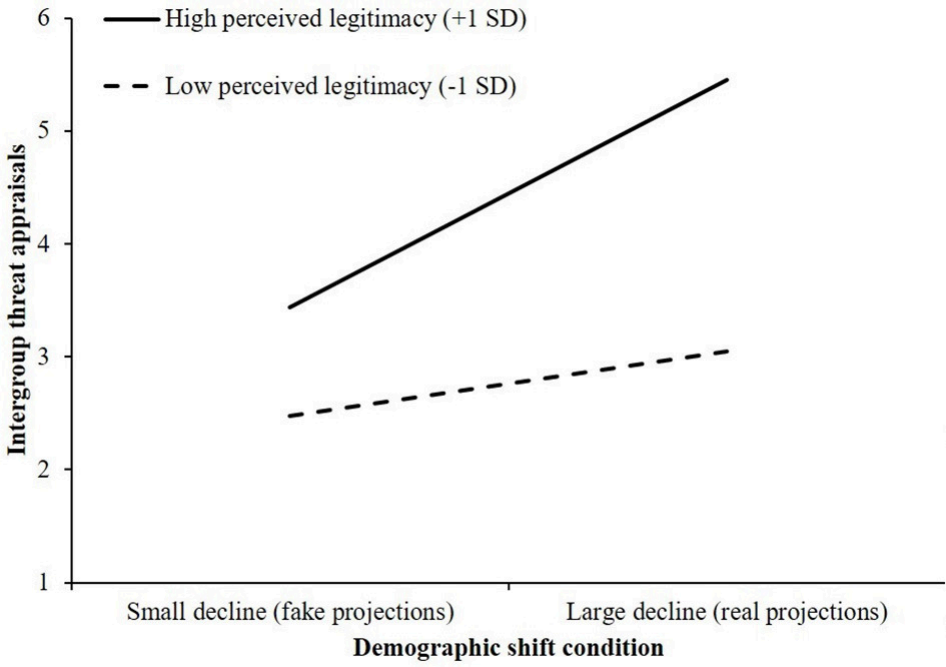
The effect of demographic shift condition and perceived legitimacy of status on intergroup threat appraisals (Study 2).

A significant interaction emerged between perceived legitimacy of status and demographic shift condition on intergroup threat appraisals, *R*^2^ch. = 0.06, *F*_(1, 88)_ = 18.26, *p* < 0.001. Inspection of the conditional effects showed that for both ethnic Portuguese people who perceived their status as highly legitimate, as well as ethnic Portuguese who saw their status as relatively illegitimate, the effect of demographic condition on the appraisal of intergroup threat was significant. As shown in Figure 4, regardless of the degree to which ethnic Portuguese people perceived their status as legitimate, viewing projections that showed large decrease in the relative size of the ethnic Portuguese population significantly heightened appraisals of intergroup threat, compared with being exposed to projections that indicated a small decline in relative size. However, the effect of seeing a large decline in the relative size of the ethnic Portuguese population on intergroup threat appraisals was stronger among those who perceived their status as highly legitimate (+1 *SD*; *B* = 2.01, *SE* = 0.23, 95% CI, [1.544, 2.475]), compared with those who perceived their status to be illegitimate (−1 *SD*; *B* = 0.57, *SE* = 0.23, 95% CI, [0.109, 1.034]).

**Figure 5:**
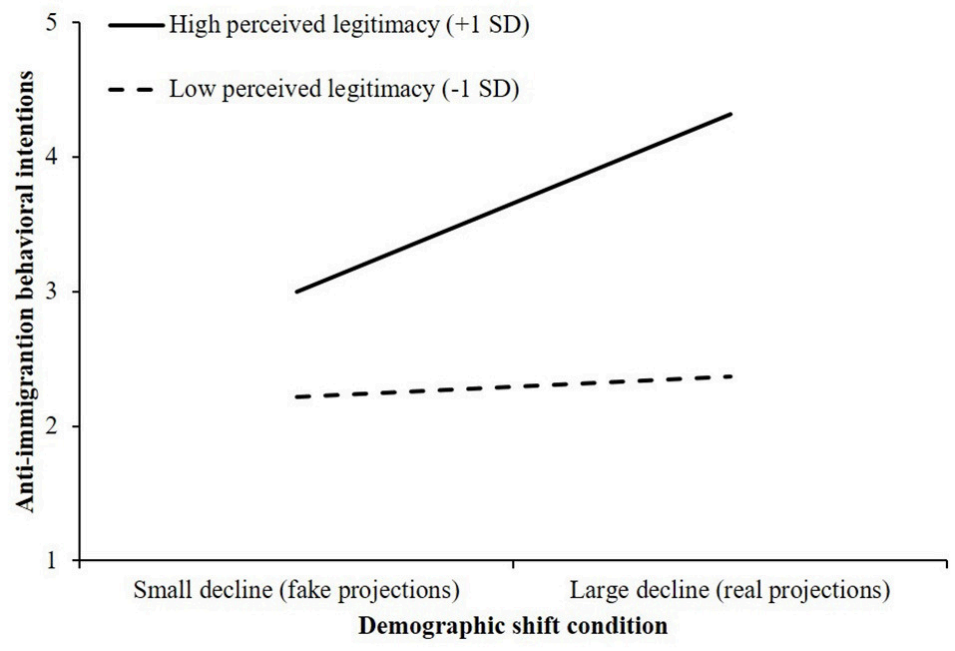
The effect of demographic shift condition and perceived legitimacy of status on anti-immigration behavioral intentions (Study 2).

The proposed interaction between perceived legitimacy of status and condition on anti-immigration behavioral intentions was significant, *R*^2^ch. = 0.04, *F*_(1, 88)_ = 6.52, *p* = 0.01. Examination of the conditional effects showed that the effect of viewing a large decline in the relative size of the ethnic Portuguese population on the anti-immigration behavioral intentions was significant for ethnic Portuguese people who perceived their status to be highly legitimate (+1 *SD*; *B* = 1.32, *SE* = 0.32, 95% CI, [0.684, 1.953]). As shown in Figure 5, for ethnic Portuguese people who viewed their status as highly legitimate, seeing a large decrease in the proportion of Portugal's population that is ethnic Portuguese population led to significantly greater willingness to engage in anti-immigration behaviors, compared with viewing a small decrease. Conversely, among ethnic Portuguese people who perceived their status as relatively illegitimate, there was no effect of condition on intention to engage in anti-immigration behaviors; intention to engage in anti-immigration behaviors was low across both conditions (−1 *SD*; *B* = 0.15, *SE* = 0.32, 95% CI, [–0.482, 0.778]).

### Discussion

The results from this study largely replicated those from the U.S. Regardless of condition, ethnic Portuguese people who appraised their status as highly legitimate expressed greater threat and reported being more willing to engage in anti-immigration behaviors compared to people who saw their status as relatively illegitimate. Those who perceived their status to be highly legitimate and were exposed to a large decline in relative size of the ethnic Portuguese population were most threatened by diversity and were most willing to engage in anti-immigration behaviors. Taken together, the findings from Portugal suggest that a nation might not need to reach a majority-minority “tipping point” for majority group members to respond negatively to demographic shifts. All it might take is a belief that one's group is deserving of their higher status.

## General discussion

Studies in North America have found that racial/ethnic majority group members tend to have negative psychological reactions to thinking about growing diversity in the future because it can signal a decline in status (e.g., Outten et al., 2012). We conducted studies in the U.S. and Portugal, and found evidence that perceiving sizeable future declines in the relative size of one's ingroup predicted heightened intergroup threat (Study 1 and 2), anger and fear toward minorities (Study 1) and also a greater willingness to engage in behaviors aimed at limiting immigration (Study 2). In accordance with SIT (Tajfel and Turner, 1979), the degree to which majority group members responded negatively to large future declines in relative size their racial/ethnic group was contingent on the degree to which they perceived their higher-status as legitimate. Specifically, the more that people believed their ingroup's higher-status was deserved, the more threatened they were by diversity and the more negatively they reacted toward minority outgroups after exposure to real projections showing a sizeable decline in the relative size of their racial/ethnic majority group.

The two studies reported here help to shed light on the psychological and contextual boundary conditions of thinking about future demographic changes. First, not all majority group members appear to be threatened by future demographic shifts, rather those who can recognize that their relative status as illegitimate do not seem as threatened by a more diverse future. This is consistent with prior studies, in that illegitimate ingroup advantages can lead members of high-status groups to feel collective guilt (Miron et al., 2006) and feelings of collective guilt can elicit favorable evaluations of relevant lower-status outgroups (Doosje et al., 1998). Regardless of condition, it was majority group members who perceived their status as legitimate who were most threatened by diversity and who reacted most negatively toward outgroups; which is consistent with the notion that perceived legitimacy makes majority group members more prone to protecting their status. However, legitimacy coupled with perceived instability—in the form of exposure to demographic projections showing a large decline in the relative size of the racial/ethnic majority population—seemed to engender the most negative responses across both cultural contexts. A finding consistent with SITs tenet that a status structure perceived as legitimate and unstable is particularly threatening for dominant groups in society (Tajfel and Turner, 1979).

Secondly, by finding a relatively similar pattern of results in Portugal we were able to demonstrate that majority group members seem to react more negatively to future demographic shifts regardless of whether or not one's group is projected to be a numerical minority in the future. This is an important finding given that Portugal is a country that differs from the U.S. on a number of cultural dimensions (Basabe and Ros, 2005; Yeganeh and May, 2011). Portugal is also a cultural context where the “tipping point”—the time when the numerical majority is expected to become a numerical minority—is much farther off. Interestingly, the percentage decrease in the relative size of the racial/ethnic majority between 2010 figures and the real projections for 2060 were nearly identical between the two studies (i.e., a 20% decrease in Portugal, from 90 to 70%; a 20.1% decrease in the United States, from 63.7 to 43.6%). Thus, it might be the case that thinking about any substantial decline in the relative size of one's ingroup can pose a threat to a majority group member's social identity.

Thirdly, it is worth noting that while greater perceived legitimacy of status was associated with increased concern over a more diverse future across both cultural contexts, this effect did appear to be stronger among the Portuguese sample (see Figure 4). Perceived legitimacy scores were also slightly higher in the Portuguese sample (see Table 2). It does make some sense, that in an older country like Portugal, where the majority group has a far greater claim to being indigenous to their nation, that ethnic Portuguese people would be more inclined to perceive their higher-status as legitimate. As such, perceptions of legitimacy of status might depend on the broader sociohistorical context and social representations of a nation's immigration history. Hence, within different countries individual ideologies should be considered in conjunction with analyses of the broader sociohistorical context.

## Limitations and future directions

Although the findings from the two studies reported here are valuable, we acknowledge that there are some limitations with this research. First, we measured rather than manipulated perceived legitimacy of status, thus making it difficult to make causal claims about perceived legitimacy of status determining reactions to anticipated demographic changes. For future studies, it would be wise to manipulate perceived legitimacy of status to confirm the moderating role of perceived legitimacy on psychological responses to future demographic shifts. Second, our independent and dependent variables were fairly obtrusive. As such, demand characteristics like social desirability might have influenced participants' responses. For example, responding to questions about how threatening demographic changes are for one's ingroup after being exposed to the respective experimental manipulations, might have led some participants to report being less threatened in an effort to appear less prejudiced. A desire to appear less prejudiced among some participants in both studies might explain why mean scores on our dependent measures fell below the midpoint (see Table 2). Past studies assessing the psychological effects of exposure to future demographic changes have also found that participants tend to score below the mean on measures of perceived threat and intergroup emotions (e.g., Outten et al., 2012; Danbold and Huo, 2015). To limit socially desirable responding, we employed deception by informing our participants that we were examining how the presentation of information affects recall ability and administering a recall test. This method of deception has been used in prior studies to limit the influence of demand characteristics (e.g., Outten et al., 2012). Future studies examining these issues might also consider directly assessing the influence of demand characteristics on participants' responses (see Rubin, 2016). A third limitation of the current research concerns the fact that our studies were not equivalent in some respects. Specifically, compared to the U.S. sample, the Portuguese sample was smaller, predominately male, and primarily comprised of people in their 20's. Thus, it might be reasonable to assume that the U.S. sample is more comparable to White American population, than the Portuguese is sample is comparable to the ethnic Portuguese population. The reason that the Portuguese sample was smaller and less varied in terms of its demographic profile was because Portuguese participants were recruited via convenience sampling on a university campus. Many of the Portuguese participants were recruited from business courses. Consequently, any generalization beyond the parameters of our study's samples must be done with a degree of caution. Some caution is also warranted with respect to the interpretation of our findings, because the dependent measures in both studies differed. While intergroup threat was measured in both studies, Study 1 also assessed negative intergroup emotions and Study 2 measured anti-immigration behavioral intentions. All of these variables constitute negative psychological reactions, but they are not identical psychological constructs. Future cross-cultural studies examining psychological reactions to future demographic changes should try to procure samples that are comparable in most respects and measure similar constructs across cultures. Taking these steps would likely strengthen the external validity of future cross-cultural investigations.

Another avenue for future study would be to further explore the cultural boundary conditions of the processes examined here. Thus far, all research in this area has been conducted in countries that currently have a White racial majority, or in the case of Portugal, a White majority ethnic group. It is important to know whether our findings would generalize to other cultural contexts. For example, countries where Whites are higher-status, but are currently a numerical minority that is expected to decline in the coming decades (e.g., South Africa), or in countries where a high-status non-White population is declining in relative size, like the United Arab Emirates (Corcoran, 2012; Malit and Al Youha, 2013).

Our findings are consistent with SIT's assumption that ingroup-favoring motivations lead members of high-status groups to act in ways that protect their high-status, assuming they see that higher status as legitimate. However, system justification theory might explain the same effects as reflecting a motivation to protect a social system that is perceived to be legitimate. Similarly, SDT might explain the effects as resulting from a motivation to maintain a hierarchy *per se*, rather than attempts to protect the ingroup's place within that hierarchy. Although these motivations are aligned when considering the perspective of dominant groups, they tend to be in conflict for members of subordinate groups. Therefore, one route for future research would be to examine how members of racial/ethnic minority groups respond to anticipated demographic changes, an area yet to be fully explored (for an exception see Craig and Richeson, 2017). From a SIT perspective (Tajfel and Turner, 1979), members of low-status groups might respond positively to increasing diversity if they perceive it as an opportunity to elevate the status of their illegitimately disadvantaged group. If low-status groups tend to perceive their lower-status as legitimate, they might not respond so positively to increasing diversity. Social dominance theory, and system justification theory, however, both point toward the possibility that members of low-status groups might actually respond to increasing diversity with hostility toward minorities, if they see that diversity as threatening a legitimate hierarchy or a just social system (Levin et al., 2002). Beyond these theoretical considerations, however, it seems important for applied and political reasons to include members of low-status groups in research on demographic change.

## Conclusion

The findings of the two studies suggest that beliefs about the security of the status hierarchy might influence how majority group members react to future demographic shifts. For those who perceive their group's current higher-status as legitimate, exposure to information about future demographic shifts seems to engender reactance in the form of being threatened by diversity and negative reactions toward minorities. Whereas individuals who can recognize that their higher-status is illegitimate to some degree are less threatened by the prospect of a more diverse future. This was true of both White Americans and ethnic Portuguese in Portugal, suggesting that the psychological underpinnings of negative reactions to greater diversity might be similar to some degree across cultural contexts. As nations become more ethnically/racially diverse, understanding the psychological and cultural boundary conditions of reactions to these demographic shifts could be crucial to managing diversity in increasingly multicultural societies.

## Ethics statement

The first study was carried out in accordance with the recommendations of the Trinity College Institutional Review Board and the Declaration of Helsinki. All participants provided electronic consent. Participants were informed of incentives for participation, their ability to withdraw from the study without penalty, the confidentiality of their responses, as well as whom they could contact if they had questions. They were are also fully debriefed after completing the study. The second study was exempt from Institutional Review. At the time the study was conceived, the Institute of Social Sciences at the University of Lisbon did not have an internal institutional review board, although the study as reported in the paper was reviewed and approved by independent scientific experts at the Fundação para a Ciência e a Tecnologia. All participants provided electronic consent in accordance with the Declaration of Helsinki and the American Psychological Association. Participants were notified of the incentives for participation, their ability to withdraw at any time without penalty, the confidentiality of their responses and whom they could contact if they had questions. All participants received a full debriefing upon completion.

## Author contributions

HO, TL, RC-L, MS, and JV contributed to the conceptualization and design of the research. HO, TL conducted data analysis and wrote the initial draft. HO, TL, RC-L, MS, and JV revised the paper.

### Conflict of interest statement

The authors declare that the research was conducted in the absence of any commercial or financial relationships that could be construed as a potential conflict of interest.

## Footnotes

^1^We also ran each of the moderation models tested in Study 1 and Study 2 while controlling for participant age and gender. The results were similar in that the conditional moderated effect of perceived legitimacy of status was still statistically significant even when controlling for age and gender. Specifically, the effects of viewing a large decline in the relative size of one's ingroup on each of the dependent measures was significant for majority group members who perceived their status to be highly legitimate, as evidenced by the 95% confidence intervals not containing zero.

## Supplementary material

### Data availability

The data supporting the conclusions of this manuscript is available upon request.
