# Supplementary figures and images for: Majority Group Members' Negative Reactions to Future Demographic Shifts Depend on the Perceived Legitimacy of Their Status: Findings from the United States and Portugal
Source: Front Psychol. 2018 Feb 13;9:79. doi: 10.3389/fpsyg.2018.00079 (PMC5816927; doi:10.3389/fpsyg.2018.00079)

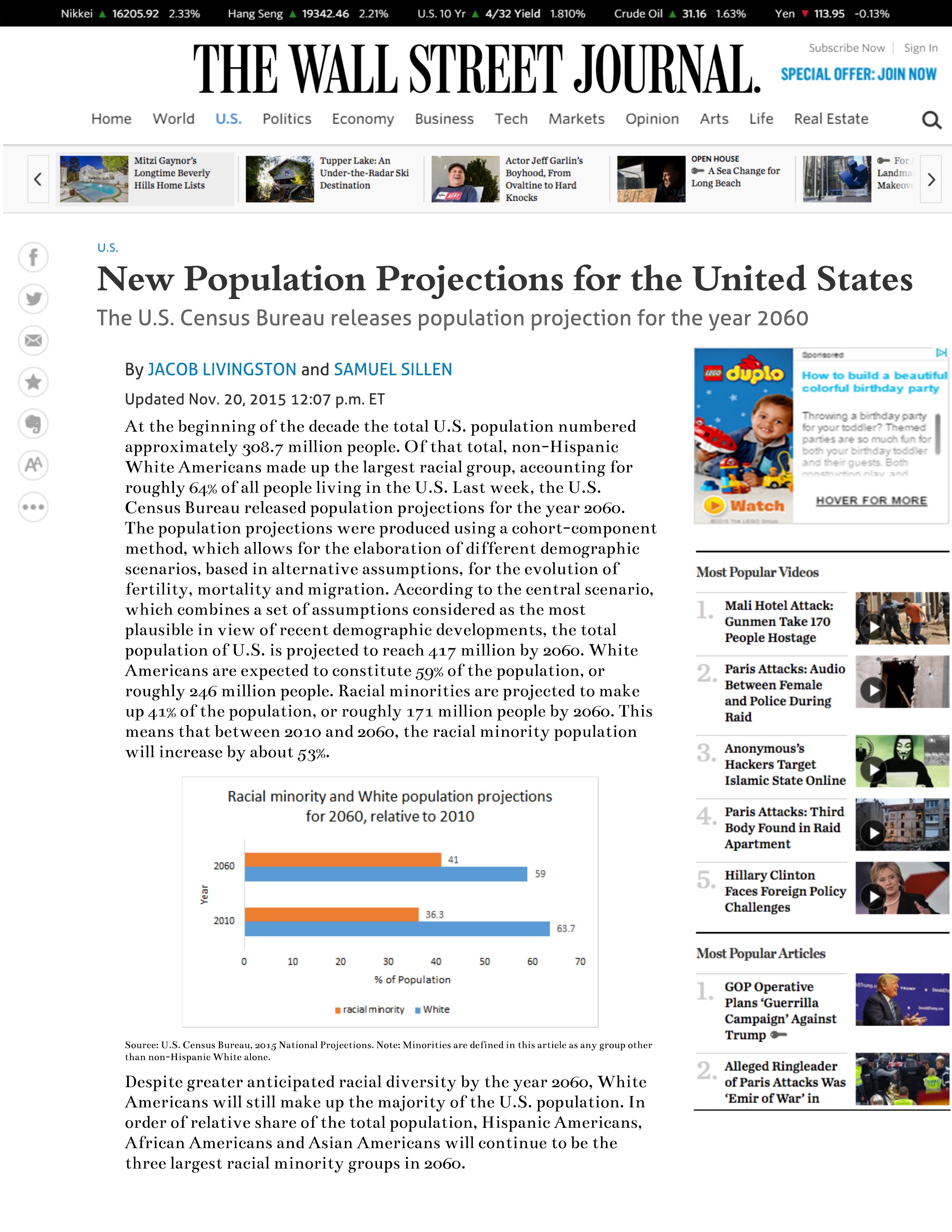

Supplement: Supplementary file 1 [file Image1.JPEG]

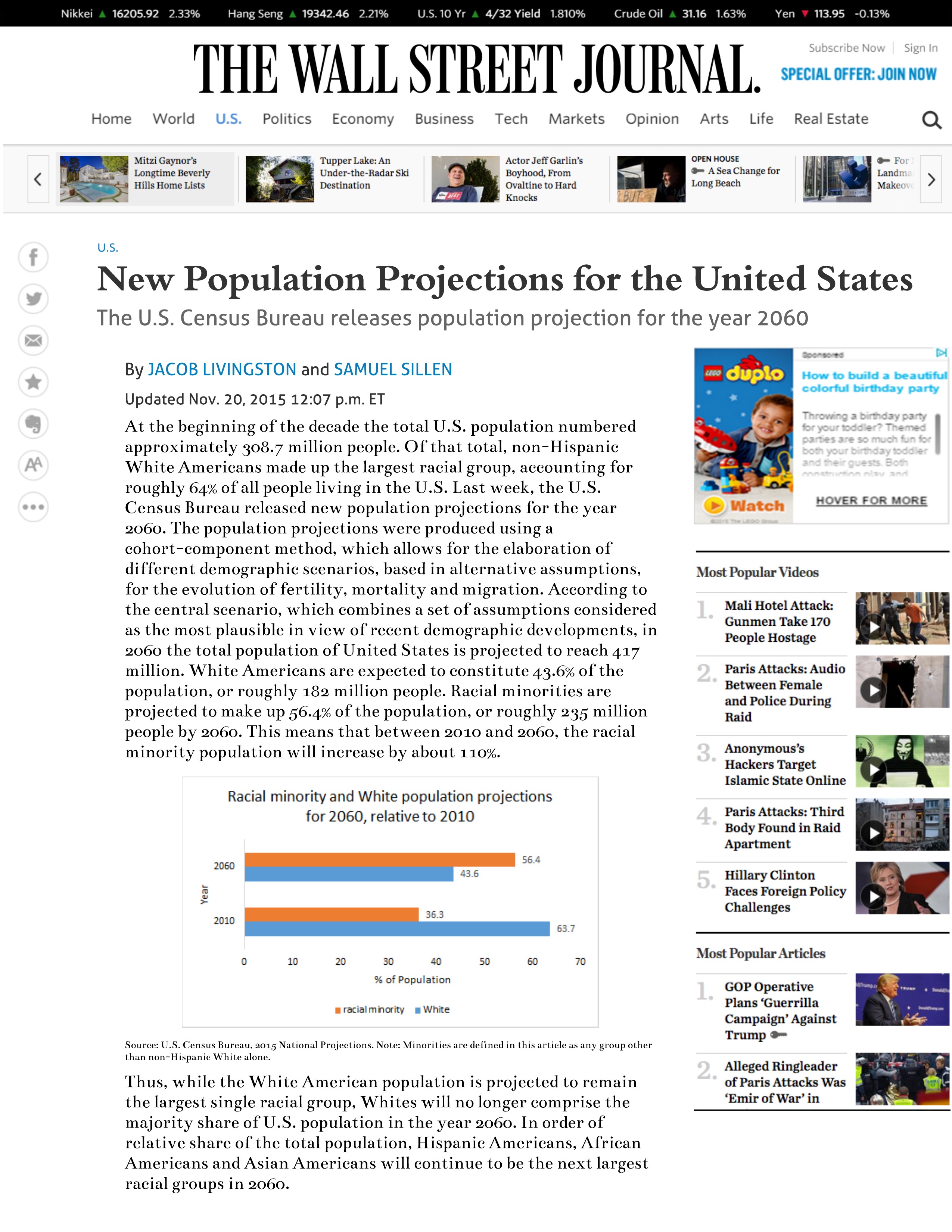

Supplement: Supplementary file 2 [file Image2.JPEG]

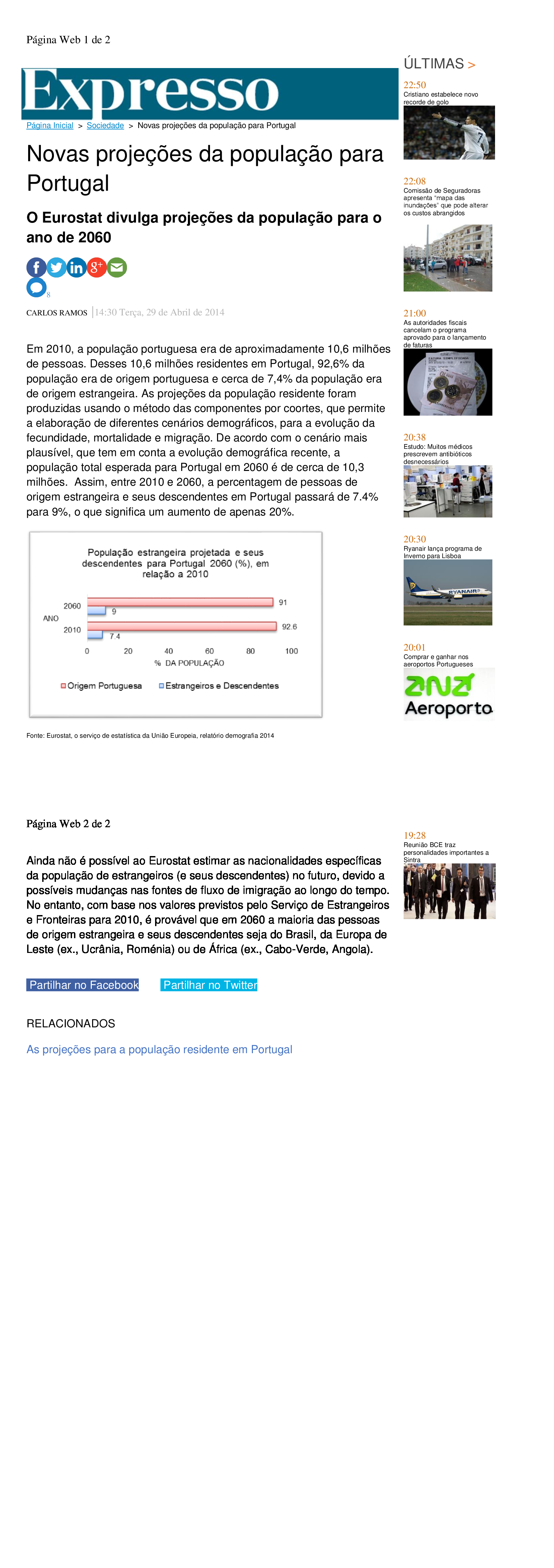

Supplement: Supplementary file 3 [file Image3.jpg]

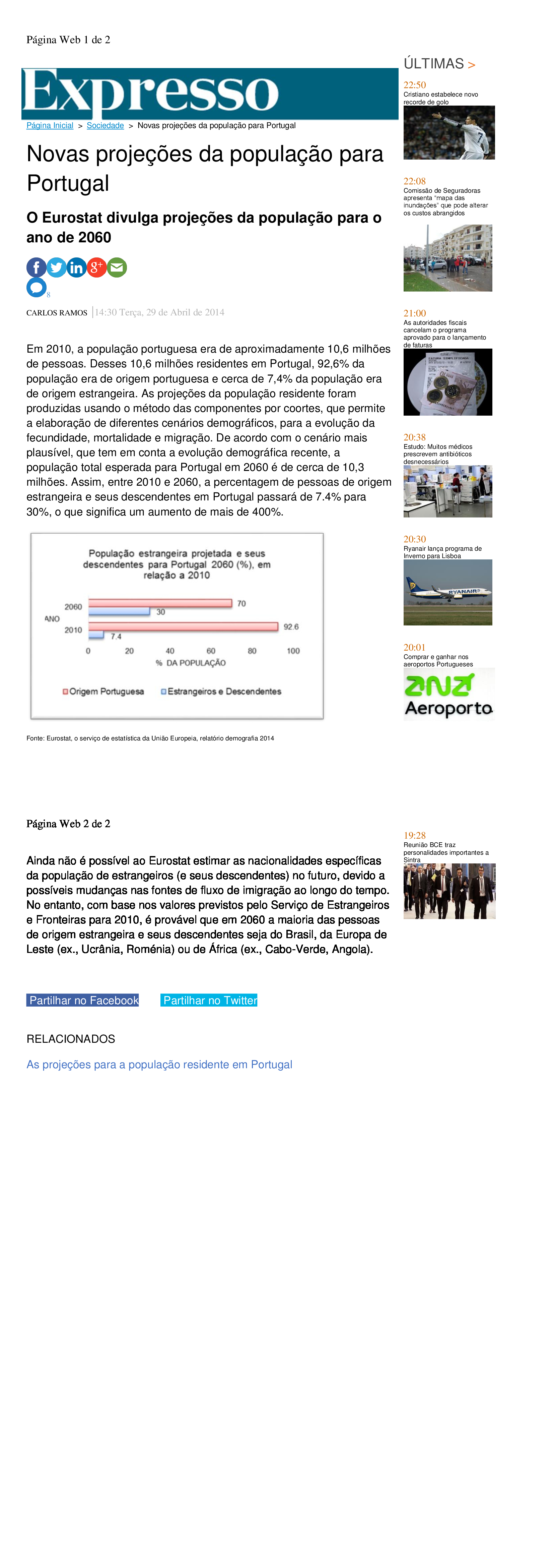

Supplement: Supplementary file 4 [file Image4.jpg]

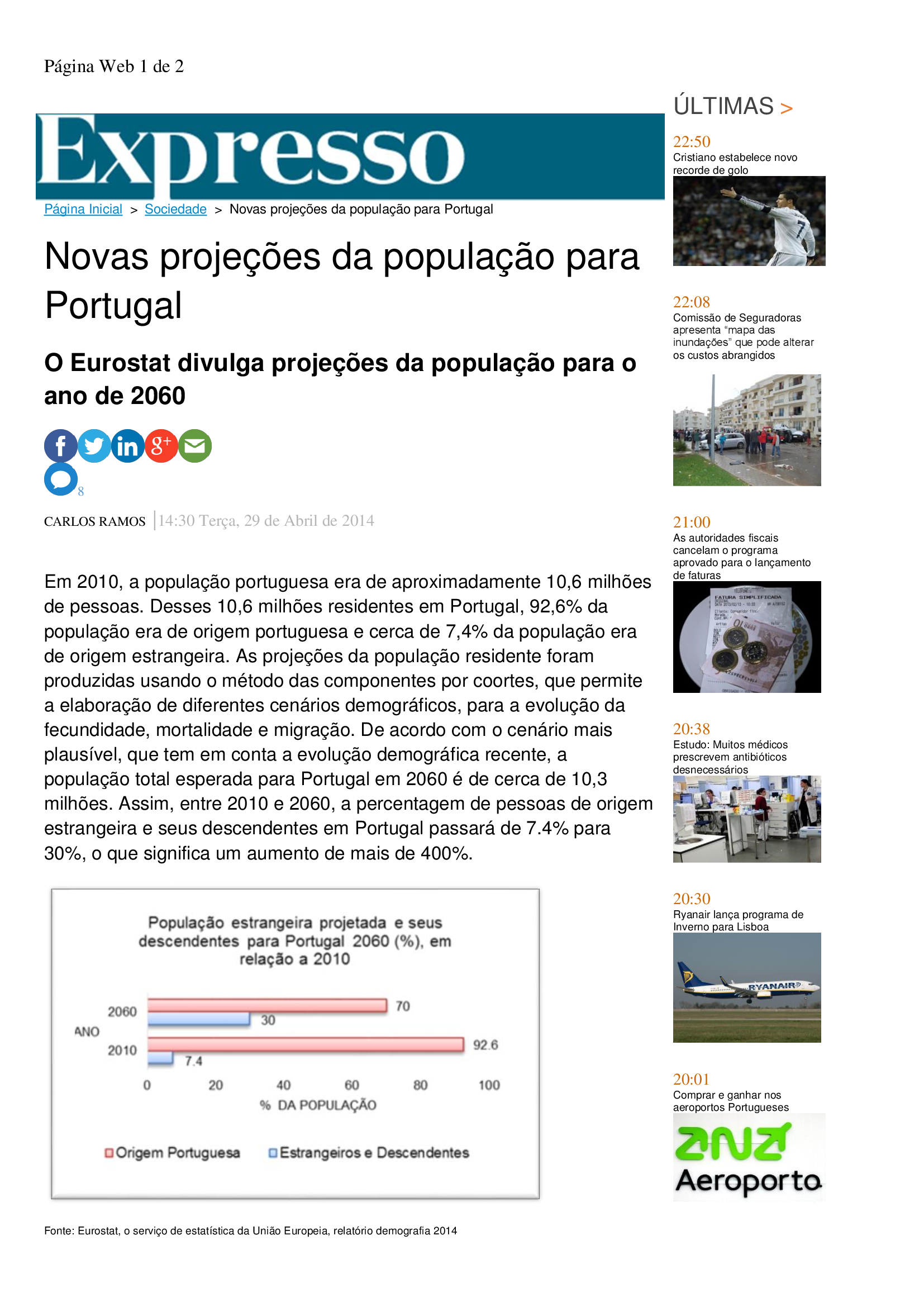

Supplement: Supplementary file 5 [file Image5.JPEG]

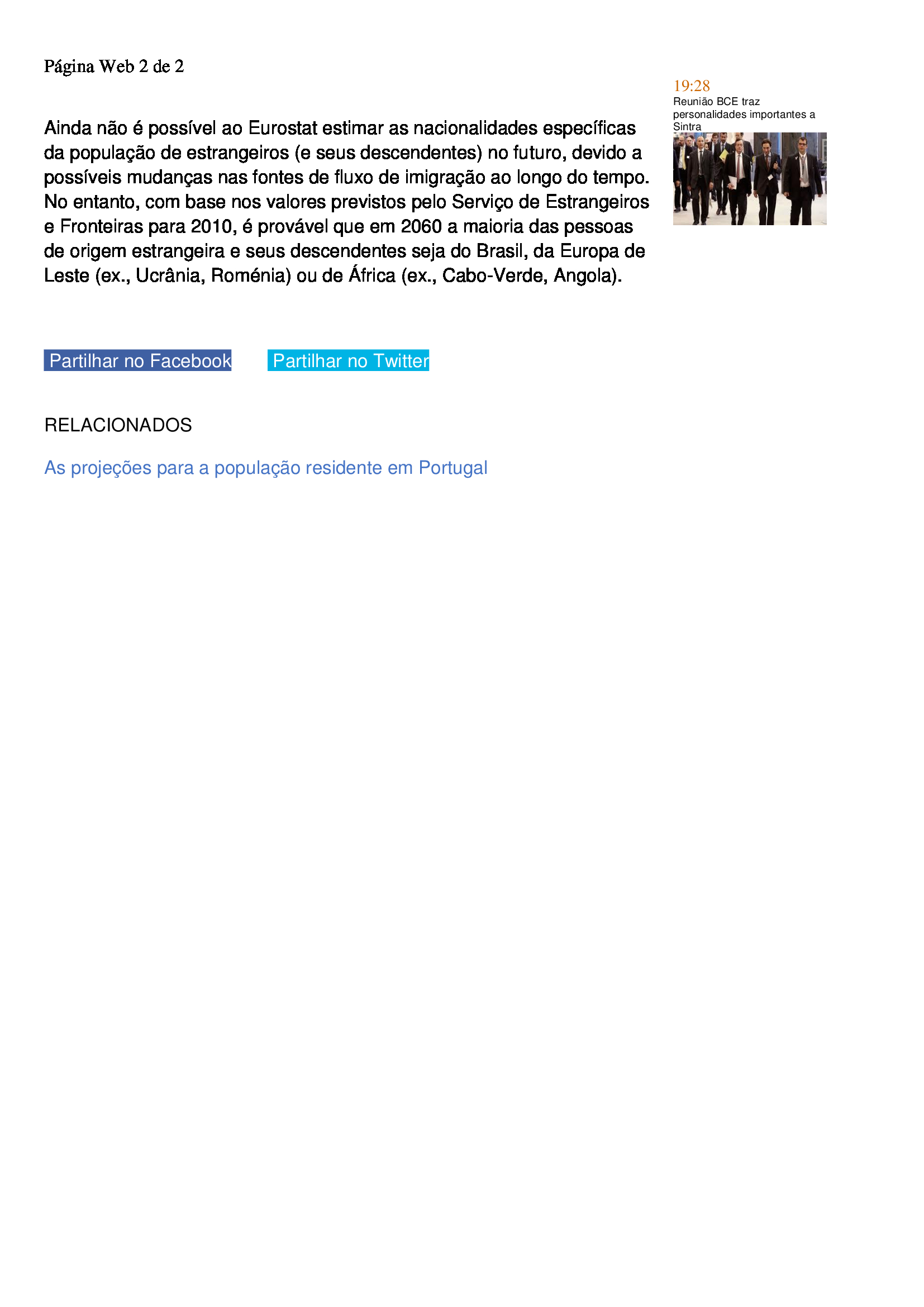

Supplement: Supplementary file 6 [file Image6.JPEG]
